# Editorial: Horizons in aging, immune system and infectious diseases

**DOI:** 10.3389/fragi.2022.1034531

**Published:** 2022-09-20

**Authors:** Christina Camell, Laura Haynes

**Affiliations:** ^1^ Dept. of Biochemistry, Institute on the Biology of Aging and Metabolism, Molecular Biology and Biophysics, University of Minnesota, Minneapolis, MN, United States; ^2^ UConn Center on Aging, University of Connecticut School of Medicine, Farmington, CT, United States

**Keywords:** immunity, aging, sex, T cells, infection, lymph node, vaccine

As the SARS-CoV-2 pandemic has reminded us, aging is one of the greatest risk factors for morbidity and mortality during infection. This Research Topic showcases articles by leaders in the field exploring some recent advances in aging, immunity and infectious diseases. There are three mini-review articles and one original research article, each of which tackles the importance of infectious diseases in older adults from a distinct angle.

The review article by Shapiro et al., examines the differences in infection and vaccine responses that are caused by sex. A female bias in immunity has been reported, but more information is needed to inform the generation of new and more effective vaccines. The authors provide insight into the importance of frailty when considering the responses of the elderly. They also provide suggestions for future studies and critiques on the data analysis that should be included.

T cell function, which is a major component of the adaptive immune response during an infection, is significantly impacted by aging. In a review article, Jin et al. have focused on the emerging concept of lysosomes in T cell immunity and the contribution of the dysfunctional lysosome to aged T cell responses. They highlight that the activation of lysosomes by mTORC1 in aged T cells may provide a therapeutic target to improve function.

In Torrelles et al. have discussed the aging microenvironment of the lung, identifying critical cellular components of protection and defining what is known about changes during aging. They describe the lung mucosa and the importance of alveolar macrophages in the protection of the lung during infection and also discuss how accumulating tissue oxidative stress is a major driver of increased susceptibility to infection in older adults.

The original research article from Kwok et al. elegantly describes changes in aged lymph node structure, including increased fibrosis which impacts T cell motility. As noted by the authors, the results of this study support a model In which the architecture of the lymph node prevents crucial FRC:lymphocyte contacts necessary for naïve T cell survival and generation of a robust immune response. This work adds to our every growing knowledge about how the aging environment impacts T cells.

We hope this Research Topic provides insight into challenges faced in the field of research on infections in older adults, while also providing stimulating insight into new upcoming areas.

